# Potential Prognostic Factors in Low Rectal Cancer Patients Treated With Neoadjuvant Chemoradiation and Abdominoperineal Resection

**DOI:** 10.7759/cureus.85171

**Published:** 2025-06-01

**Authors:** Mohamed I Fahim, Fady Shafeik, Rasha M Allam, Marina Asaad, Mohammad Taher

**Affiliations:** 1 Surgical Oncology, National Cancer Institute - Cairo University, Cairo, EGY; 2 Oncosurgery, National Cancer Institute, Cairo, EGY; 3 Epidemiology and Biostatistics, National Cancer Institute - Cairo University, Cairo, EGY; 4 Pediatrics, Cairo University Hospital, Cairo, EGY

**Keywords:** abdominoperineal resection, conventional abdominoperineal resection, low rectal cancer, neoadjuvant chemoradiation, neoadjuvant chemo radiation

## Abstract

Aim

This study aimed to evaluate the potential prognostic impact of various clinicopathological factors on survival outcomes in patients with low rectal adenocarcinoma treated with neoadjuvant chemoradiation followed by abdominoperineal resection.

Methods

This retrospective observational cohort study included 174 patients with low rectal adenocarcinoma who were treated and followed up between 2012 and 2019 at the National Cancer Institute - Cairo University.

Results

The median follow-up period was 71.2 months. The median disease-free survival (DFS) was 69 months, while the median overall survival (OS) was not reached. Multivariate analysis showed that high tumor grade was significantly associated with reduced OS (95% CI: 1.250-7.280; P = 0.014). Additionally, a circumferential resection margin (CRM) of ≤1 mm was significantly associated with reduced DFS (95% CI: 1.604-17.818; P = 0.006).

Conclusions

The study found no significant prognostic impact of tumor response to neoadjuvant chemoradiation. However, tumor grade and CRM status emerged as potential prognostic factors for survival in this patient population.

## Introduction

For patients with locally advanced mid and low rectal cancer, the standard treatment approach typically consists of neoadjuvant chemoradiotherapy followed by surgical resection [1-3]. This approach aims to reduce tumor size, enhance surgical margins, and decrease the risk of local recurrence [4-6].

Despite this established strategy, there is ongoing debate about which clinicopathological factors most significantly influence long-term outcomes in these patients [7-9].

This study aims to assess the prognostic significance of the pathological response to neoadjuvant chemoradiotherapy, along with other clinical and histological variables, in relation to overall survival (OS) and disease-free survival (DFS) in patients with low rectal adenocarcinoma undergoing abdominoperineal resection (APR).

## Materials and methods

Study design

This retrospective cohort study was conducted at the National Cancer Institute (NCI) - Cairo University between 2012 and 2019.

Study population and sample size

A total of 174 patients diagnosed with low rectal adenocarcinoma were included. Eligible participants were adults with good performance status, preserved hepatic and renal function, and no evidence of metastatic disease. All patients received neoadjuvant chemoradiotherapy followed by APR. Patients were excluded if they experienced disease progression during neoadjuvant treatment, developed significant postoperative complications, or were in poor general condition.

Treatment protocol

All patients underwent APR with permanent colostomy, performed six to eight weeks after completing neoadjuvant treatment. MRI of the abdomen and pelvis was conducted prior to neoadjuvant therapy and again six weeks after its completion, before surgery.

Postoperative follow-up

Following hospital discharge, patients were monitored according to a structured follow-up schedule beginning three months postoperatively and continuing every six months. Follow-up evaluations included CT scans of the chest, abdomen, and pelvis, along with laboratory tests such as serum CA 19-9, carcinoembryonic antigen, complete blood count, and assessments of renal and hepatic function.

Study measures

The pathological response to neoadjuvant therapy was assessed using the tumor regression grade (TRG) system. TRG was classified as follows: Grade 0 indicated a complete response, with no viable tumor cells; Grade 1 represented a marked response, with minimal residual tumor; Grade 2 denoted a moderate response, where the tumor was outgrown by fibrosis; and Grade 3 reflected a poor or no response, characterized by extensive residual disease [4].

OS was defined as the time from initial diagnosis to the last recorded follow-up in which the patient was alive. DFS was measured from the date of surgery to the occurrence of either recurrence or death, whichever came first.

Ethical consideration

The study protocol was approved by the Institutional Review Board of the NCI - Cairo University. Patient data were anonymized, and confidentiality was maintained throughout the study. As this was a retrospective study, the requirement for informed consent was waived.

Statistical analysis

All data were analyzed using IBM SPSS Statistics for Windows, Version 23.0 (Released 2015; IBM Corp., Armonk, NY, USA). Continuous variables were summarized as means, medians, and ranges, while categorical variables were presented as frequencies and percentages. Survival outcomes were estimated using the Kaplan-Meier method, and differences between survival curves were assessed with the log-rank test. Multivariate analysis was performed using the Cox regression model to evaluate the independent prognostic value of variables that were statistically significant in univariate analysis. HRs and 95% CIs were calculated. A P value ≤ 0.05 was considered statistically significant. All statistical tests were two-tailed.

## Results

**Figure 1:**
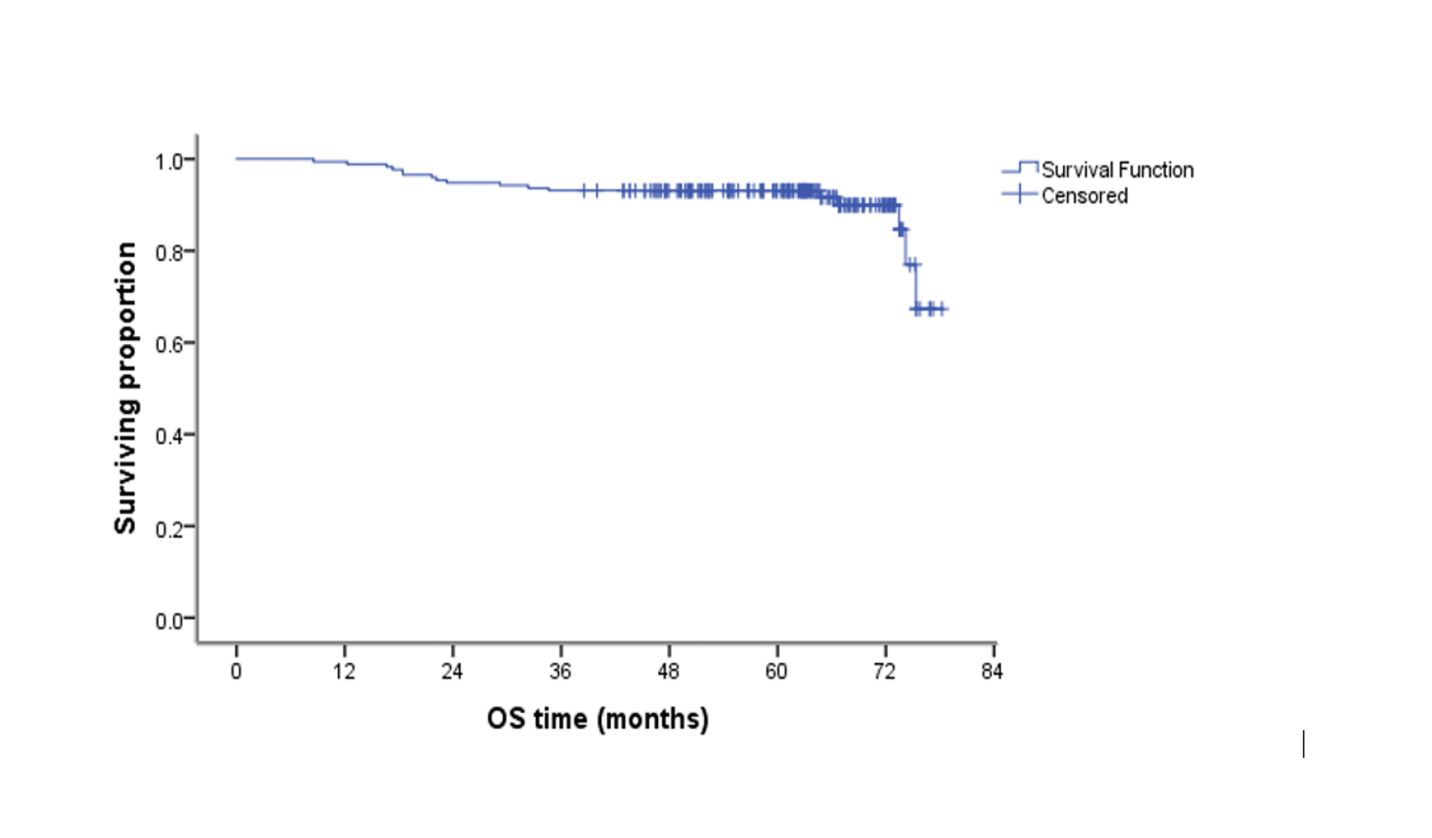
Kaplan-Meier curve showing OS The median OS was not reached. OS, overall survival

**Figure 2:**
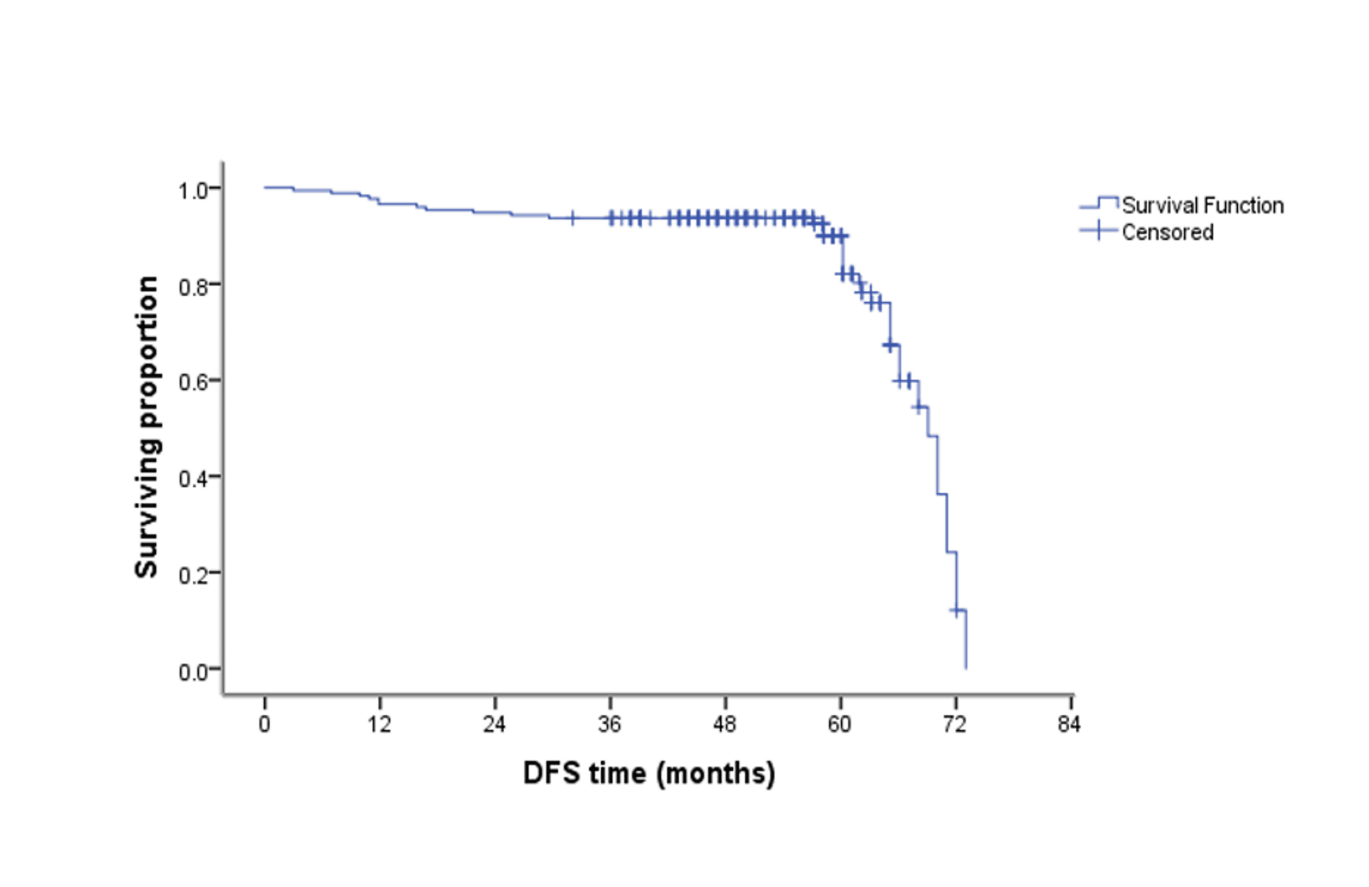
Kaplan-Meier curve showing DFS The median DFS was 69 months. DFS, disease-free survival

A total of 174 patients with low rectal adenocarcinoma were treated and followed up in this study. The median age was 50 years, with a range of 21 to 82 years. The clinical and tumor characteristics of the patients are summarized in Table 1. The surgical procedures lasted between four and five hours, and patients were typically discharged within 10 days postoperatively. The median follow-up period was 71.2 months. The median DFS was 69 months; however, the median OS was not reached (Figure 1, Figure 2).

**Table 1:** Patient and tumor characteristics (N = 174) CRM, circumferential resection margin; TRG, tumor regression grade

Characteristics	N (%)
Age	
≤50 years	128 (73.56%)
>50 years	46 (26.44%)
Sex	
Male	69 (39.66%)
Female	105 (60.34%)
Grade	
Non-high grade (low and intermediate)	110 (63.22%)
High grade	64 (36.78%)
Radiological T stage before neoadjuvant treatment	
T1	0
T2	6 (3.45%)
T3	164 (94.25%)
T4	4 (2.3%)
Radiological T stage after neoadjuvant treatment	
T0	6 (3.45%)
T1	3 (1.72%)
T2	99 (56.9%)
T3	66 (37.93%)
T4	0
Pathological T stage	
T0	9 (5.17%)
T1	6 (3.45%)
T2	39 (22.41%)
T3	111 (63.79%)
T4	9 (5.17%)
Radiological N stage before neoadjuvant treatment	
N0	51 (29.31%)
N1	66 (37.93%)
N2	57 (32.76%)
Radiological N stage after neoadjuvant treatment	
N0	99 (56.9%)
N1	48 (27.59%)
N2	27 (15.52%)
Pathological N stage	
N0	103 (59.2%)
N1	36 (20.69%)
N2	35 (20.11%)
Therapy effect (TRG)	
Grade 0	18 (10.34%)
Grade 1	33 (18.97%)
Grade 2	53 (30.46%)
Grade 3	70 (40.23%)
CRM	
≤1 mm	99 (56.9%)
>1 mm	75 (43.1%)

Univariate analysis of various patient and tumor characteristics revealed that tumor grade (P < 0.001), radiological N stage before neoadjuvant treatment (P = 0.016), and pathological N stage (P = 0.002) were significantly associated with OS. However, in the multivariate analysis, only high-grade tumors remained significantly associated with reduced OS (95% CI: 1.250-7.280, P = 0.014).

For DFS, univariate analysis indicated that tumor grade (P = 0.004) and CRM (P = 0.002) had a statistically significant impact. Multivariate analysis identified a CRM of ≤1 mm as the only factor significantly associated with reduced DFS (95% CI: 1.604-17.818, P = 0.006).

## Discussion

While neoadjuvant chemoradiotherapy is well recognized for facilitating tumor downstaging and reducing circumferential resection margin (CRM) positivity [2,3], this study found no significant survival benefit based on TRG. This suggests that achieving negative surgical margins may be more important than the extent of tumor shrinkage itself, especially in low rectal tumors where surgical access and margin clearance pose technical challenges.

Our findings indicate that the pathological response to neoadjuvant chemoradiotherapy did not have a significant prognostic effect on OS or DFS. This contrasts with previous research involving 385 rectal cancer patients, which demonstrated that the degree of tumor regression after preoperative chemoradiation was a meaningful predictor of patient outcomes [5].

Similarly, a study of 342 patients with locally advanced rectal cancer who underwent preoperative chemoradiation concluded that tumor response to treatment significantly impacts DFS, while the final pathological stage after surgery also influences outcomes [6]. Another study including 237 rectal cancer patients found that both TRG and lymph node stage had prognostic value for survival [7]. However, in the present study, no statistically significant prognostic impact of pathological stage or therapy effect was observed.

High tumor grade emerged as the sole independent predictor of reduced OS in this cohort. This finding is consistent with prior reports linking poorly differentiated tumors to more aggressive clinical behavior and poorer treatment response [7,9]. Tumor grade thus remains a simple yet powerful prognostic factor that could help guide decisions regarding closer surveillance or more aggressive adjuvant therapy.

Although lymph node involvement showed significance on univariate analysis, it did not retain independent prognostic value in multivariate models. This contrasts with studies such as Huebner et al. [7], who identified nodal status as one of the strongest predictors of survival after chemoradiotherapy. Differences in staging accuracy, lymph node yield during surgery, or pathological assessment standards may account for these discrepancies.

The prognostic significance of the CRM is widely recognized, with previous studies demonstrating its impact on local recurrence, distant metastasis, and survival [8]. In this study, univariate analysis showed that CRM status significantly influenced both OS and DFS. However, multivariate analysis revealed its independent prognostic significance only for DFS.

Supporting this, a study of 146 rectal cancer patients treated with preoperative radiation who had residual disease reported that CRM status significantly affected both recurrence-free and cancer-specific survival [9].

This study has some limitations, including a relatively small sample size of 174 patients. Larger studies with longer follow-up could yield more definitive results. Additionally, as a retrospective cohort study without standardized patient selection criteria (e.g., variable responses to neoadjuvant therapy and tumor stages) and with surgeries performed by multiple surgeons, these factors may contribute to the relatively high proportion of cases with CRM ≤1 mm.

Emerging technologies, such as deep learning, have shown promise in enhancing colorectal cancer diagnostics, particularly through the analysis of histopathology images. Convolutional neural networks and similar algorithms can improve the accuracy and speed of tissue evaluation, potentially supporting clinical decision-making [10,11]. While this study focused on traditional clinicopathological factors, such innovations may play an important role in developing personalized management strategies for colorectal cancer in the future.

## Conclusions

Our multivariate analysis identified high tumor grade as an independent predictor of reduced OS and CRM involvement (≤1 mm) as a significant factor negatively impacting DFS. Although neoadjuvant chemoradiotherapy aids tumor downstaging and facilitates margin-negative resections, the degree of pathological tumor regression following this treatment did not independently influence long-term survival outcomes in this patient cohort.
